# Combating muscle atrophy: emerging therapeutic targets that are fiber‐type‐specific

**DOI:** 10.1111/febs.70241

**Published:** 2025-08-28

**Authors:** Samrat Chakraborty, Raz Ben‐David, Shenhav Shemer

**Affiliations:** ^1^ Faculty of Biology Technion Institute of Technology Haifa Israel

**Keywords:** fiber type, muscle atrophy, myosin, skeletal muscle, therapeutic targets

## Abstract

Skeletal muscle is essential for life as it enables physical movement, maintains posture, is crucial for breathing, and serves as a major site for energy and carbohydrate metabolism. Pathological conditions that reduce skeletal muscle mass and function—such as muscular dystrophies, motor‐neuron diseases, cancer, type‐2 diabetes, or aging—have detrimental effects on human health, reducing quality of life and survival. Currently, exercise is the only validated treatment for increasing muscle mass and function, but it is impractical for bedridden patients or the frail elderly. Significant advances in understanding the molecular mechanisms underlying atrophy of slow‐ or fast‐twitch muscle fibers have identified numerous previously unknown key players that may show promise as potential drug targets. Here, we review these recent advances and discuss the potential of these discovered mechanisms as therapeutic targets to combat muscle wasting.

## Graphical abstract

Inflammation, denervation or decreased insulin signaling activate proteolysis and cause muscle wasting. Pathological conditions like obesity and DMD can also exacerbate atrophy via PGD_2_–RhoA–ROCK2 signaling. In contrast, exercise, dietary interventions, and pharmacological agents can activate Ca^2+^/calcineurin–NFAT and AMPK–PGC‐1α–MEF pathways, promoting reprogramming to slow‐twitch fibers, which are more resistant to atrophy. Targeting these pathways may attenuate atrophy and preserve muscle function. Created with BioRender.

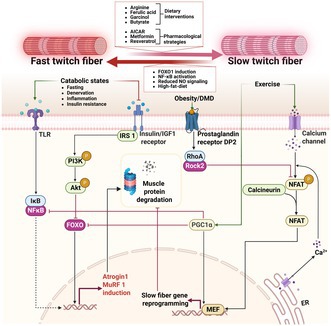

**Abbreviations**
AICAR: 5‐aminoimidazole‐4‐carboxamide riboside
ALS: amyotrophic lateral sclerosis
AMPK: AMP‐activated protein kinase
CaMK: Calcium/calmodulin‐dependent protein kinase
cIAP1: cellular inhibitor of apoptosis 1
DMD: Duchenne muscular dystrophy
eEF2K: eukaryotic elongation factor 2 kinase
eIF3‐f: eukaryotic translation initiation factor 3 subunit f
FA: ferulic acid
HDAC4: histone deacetylase 4
HFD: high‐fat diet
LDH: lactate dehydrogenase
MDH: malate dehydrogenase
MEF2: myocyte enhancer factor 2
MRF: myogenic regulatory factor
MuRF1: muscle‐specific RING Finger 1
MYH: myosin heavy chain
MYOD1: myogenic differentiation 1
NFAT: nuclear Factor of Activated T‐cells
nNOS: neuronal nitric oxide synthase
NO: nitric oxide
p38 MAPK: p38 mitogen‐activated protein kinase
PGC‐1α: peroxisome proliferator‐activated receptor‐γ coactivator‐1α
PGD2: prostaglandin D
PPAR: peroxisome proliferator‐activated receptor
SDH: succinate dehydrogenase
SERCA: Sarcoplasmic/Endoplasmic Reticulum Ca^2+^‐ATPase
SIRT1: sirtuin 1
SOX6: SRY‐Box transcription factor 6
TCA: tricarboxylic acid cycle
TRAF6: tumor necrosis factor receptor‐associated factor 6
TRPV1: transient receptor potential vanilloid 1
TSC2: tuberous sclerosis complex 2
TWEAK: tumor necrosis factor‐like weak inducer of apoptosis
ULK1: uncoordinated‐51‐like kinase 1
UPS: ubiquitin‐proteasome system

## Introduction

Skeletal muscle is essential for various physiological functions, as it drives physical movement and serves as a major pillar of metabolism. This tissue comprises approximately 40% of total human body weight and 50% of the body's protein content. Disuse of skeletal muscle, resulting from conditions such as spinal cord injuries, amyotrophic lateral sclerosis (ALS), Duchenne muscular dystrophy (DMD), prolonged immobilization in bedridden or ventilated patients, as well as systemic catabolic conditions like cancer, aging, obesity, type‐2 diabetes, or starvation, disrupts the delicate balance between rates of protein synthesis and degradation, ultimately leading to excessive protein breakdown and loss of muscle mass and strength. The resulting skeletal muscle atrophy, if prolonged, causes weakness, frailty, disability, impaired systemic metabolism, delays in recovery from acute illness and injury, and promotes morbidity and mortality. Seminal research over the past few decades has significantly advanced our understanding of the cellular mechanisms driving muscle wasting. Despite this progress, there remains an urgent and unmet medical need for effective, approved therapies to combat atrophy.

Muscle fiber types respond differently to conditions that induce atrophy, such as muscular dystrophies, disuse, aging, or disease (Table 1), providing insights into potential therapeutic strategies. Fast‐twitch fibers are generally more susceptible to atrophy, whereas slow‐twitch fibers tend to better maintain their size in catabolic conditions like aging [1]. Exercise‐induced mechanisms that shift muscle fibers toward slow‐twitch phenotype have been shown to provide broad protection against various forms of muscle wasting. However, it is critical to recognize that preserving fast‐twitch fibers is essential for overall health and strength, as they play a key role in preventing muscle wasting and maintaining functional strength. In this review, we summarize emerging insights into the specific mechanisms underlying atrophy of specific muscle fiber types and highlight key players that can be therapeutically targeted to treat or prevent atrophy.

**Table 1:** Fiber‐type transitions in muscle atrophy.

Atrophy condition	Affected fiber type	Fiber‐type transition	Key factors contributing to fiber atrophy
Aging (sarcopenia)	Preferential loss of Type II fibers	Type II → Type I	↑ Atrogenes ↓ IGF‐1 ↑ Inflammation
Disuse (immobilization, bed rest)	Type I and II fibers	Type I → Type II	↓ AMPK ↓ mTORC1 ↑ NF‐κB ↑ FOXO3 ↓ NO
Denervation	Type I and II fibers	Type I → Type II	↓ Calcineurin/NFAT ↑ NF‐κB
Obesity	Significant loss of Type I	Type I → Type II	↓ Insulin sensitivity ↑ PGD2
Spaceflight/microgravity	Preferential loss of Type I	Type I → Type II	↓ Calcineurin/NFAT ↓ AMPK ↓ PGC‐1a

## Skeletal muscle fiber types

Skeletal muscle consists of diverse fiber types, whose distinct characteristics are established during embryonic development by intrinsic myogenic mechanisms. The relative distribution of these fiber types varies across species and among individuals in humans [2]. This diversity enables muscles to adapt to a wide range of functional demands and physiological changes. For example, the presence of different fiber types within a single muscle enables the same muscle to perform multiple roles, from maintaining posture to generating powerful, rapid movements. Mammalian skeletal muscle fibers are classified based on their contractile rates, energy metabolism profiles, and the expression of distinct myosin isoforms, all of which ensure optimal performance for specific physiological demands, from high‐intensity movements to sustained endurance activities [3].

In adult mammalian muscle, fibers are typically categorized into type I (slow‐twitch) and type II (fast‐twitch), while type II fibers are further divided into types IIA, IIB, and IIX, based on the expression of distinct myosin heavy chain (MYH) isoforms [2]. These MYH isoforms are associated with contractile velocity and metabolic properties of individual muscle fibers, rendering them more suited for rapid, powerful contractions (fast‐twitch fibers) or sustained endurance activities (slow‐twitch fibers). Slow‐twitch muscle fibers rely on oxidative metabolism, exhibit high fatigue resistance, and endure contraction but produce less force and velocity than the other fiber types. In contrast, fast‐twitch fibers primarily depend on glycolytic metabolism to generate rapid, powerful contraction, but they are more susceptible to fatigue [4]. The cross‐sectional area of muscle fiber types differs between species. In humans, type I (slow‐twitch) and type II (fast‐twitch) fibers are generally similar in size [1, 5]. However, in laboratory rodents such as mice and rats, fast‐twitch fibers, particularly type IIB, are significantly larger than slow‐twitch fibers, sometimes by more than twofold [5]. These interspecies differences in muscle fiber size and composition, particularly the predominance and larger size of type IIB fibers in rodents, should be carefully considered when interpreting preclinical studies of therapies for muscle wasting. Consequently, assessments of fiber‐type switching under catabolic conditions or as a therapeutic strategy should be validated in humans using additional parameters beyond fiber size alone to ensure clinical applicability. Besides fibers predominantly expressing a single MYH isoform, muscles also contain fibers that express two or more distinct MYH isoforms, known as hybrid fibers. These hybrid fibers create additional subtypes, such as I/IIA, IIA/IIX, and IIX/IIB, contributing to a nearly continuous range of ATP consumption and contraction speeds, from the fastest type IIB fibers to the slowest type I fibers [6]. All these fiber types have been identified in murine and other mammalian species; however, in humans, muscle fiber types I, IIA, and IIX predominate (Table 2) [7, 8].

**Table 2:** Properties of skeletal muscle fiber types in mammals.

Fiber type	Myosin isoform	Contractile velocity	Fatigue resistance	Metabolism
Type I (slow)	MYH 7	Slow	High	Oxidative
Type IIA (fast)	MYH 2	Fast	Moderate	Oxidative
Type IIX (fast)	MYH 4	Very fast	Low	Glycolytic
Type IIB (fast)	MYH 1	Very fast	Low	Glycolytic

### Transcriptional regulation of fiber‐type identity

In addition to MYH expression, fiber‐type identities are determined by sarcomeric components, such as fast and slow tropomyosin isoforms, and regulatory elements like fast and slow Sarcoplasmic/Endoplasmic Reticulum Ca^2+^‐ATPase (SERCA) isoforms. Transcription factors like Myogenic Differentiation 1 (MYOD1), which belongs to the myogenic regulatory factor (MRF) family, and SRY‐Box Transcription Factor 6 (SOX6), promote the differentiation to fast‐twitch fibers [9, 10, 11]. Conversely, the transcription factor Nuclear Factor of Activated T‐cells (NFAT), particularly NFATc1, together with Myocyte Enhancer Factor 2 (MEF2), promotes the expression of slow‐twitch‐specific genes [12, 13]. To date, the precise molecular mechanisms by which physiological catabolic cues trigger transitions between fiber types remain uncertain. Understanding how these molecular pathways are activated could lead to the development of therapies that target specific fiber types to prevent or treat atrophy.

### Functional differences and metabolic characteristics

Slow‐twitch fibers rely on aerobic respiration, which is slower than glycolytic metabolism, but more efficient at producing ATP. This slower, steady supply of ATP limits the rate of cross‐bridge (myosin binding to actin) cycling, resulting in relatively slow and prolonged contraction. These fibers are rich in mitochondria [14], have an extensive capillary supply, and high myoglobin content to enhance oxygen delivery to muscle and render these fibers their characteristic red color. These features allow slow‐twitch fibers to sustain low‐intensity contractions without fatigue [15], making them suitable for endurance activities such as long runs, as well as generation of isometric contractions to maintain posture. However, slow‐twitch fibers generate less force compared with fast‐twitch fibers.

Fast‐twitch fiber types IIX and IIB predominantly utilize glycolytic metabolism, which provides a rapid but less efficient supply of ATP, supporting powerful contractions but also faster fatigue. These fibers contain fewer mitochondria, reduced capillary supply, and are optimized for high‐intensity and short‐duration movement such as sprinting, jumping, and weightlifting [2]. Importantly, these fast‐twitch fibers are critical for preventing muscle wasting, as they contribute to maintaining overall muscle strength and function. The cellular decision to switch between glycolytic or oxidative metabolism allows the muscle to meet the energy demands of different activities. During strenuous exertion, the shift from oxidative phosphorylation to anaerobic glycolysis supports a rapid increase in ATP production, which is crucial for maintaining performance under stress.

## Foundational concepts of muscle atrophy

Whether a muscle grows or atrophy, it significantly influences the body's ability to move, breathe, and maintain systemic metabolism. Atrophy can be a prognostic marker for various pathological conditions, ranging from organ dysfunction to premature aging or cancer [16, 17]. Under normal conditions, muscle mass is maintained by balanced rates of protein synthesis and degradation. However, during atrophy, this balance shifts toward increased proteolysis [18]. The degradation of muscle proteins, particularly the contractile myofibrils, occurs primarily via the proteasome, and their loss accounts for the decline in muscle mass and strength. Additionally, mitochondria and other organelles are degraded via autophagy, which contributes to the reduced endurance capacity of atrophying muscles.

Loss of muscle mass due to disuse (e.g., bedridden or ventilated patients, immobilization, and microgravity in space), aging, malnutrition, denervation (e.g., spinal cord injuries, motor‐neuron diseases), or cancer‐induced cachexia is characterized by transcriptional [19, 20, 21] and metabolic [22] remodeling. Atrophying muscle fibers exhibit a smaller cross‐sectional area compared with normal fibers, mainly due to the accelerated degradation of myofibrillar proteins that normally occupy most of the volume of the cell. These atrophying fibers typically do not undergo apoptosis and retain most of the structural features of a normal skeletal muscle [23]. Therefore, muscle atrophy is not a chaotic process but rather occurs in a controlled, gradual, and regulated manner, allowing muscles to sustain contraction even under conditions of rapid wasting (e.g., in fasting or cancer) [24].

The ordered process of atrophy involves a bi‐phasic induction of genes that promote a gradual disassembly and loss of the contractile myofibrils. Early transcriptional profiling of atrophying muscles identified a subset of genes, termed atrogenes, that are similarly induced or repressed across various catabolic conditions. These include E1, E2s, E3s, ubiquitin, proteasome subunits, and factors that mediate autophagy [19, 21, 25, 26]. Recent genome‐wide RNA sequencing has revealed that these genes are induced in two distinct phases during atrophy, early and late [27, 28]. Two ubiquitin ligases, muscle‐specific RING Finger 1 (MuRF1) and atrogin1/MAFbx, are markedly induced in most types of atrophy and are widely recognized as bona fide atrophy markers [25]. MuRF1 is responsible for ubiquitination and degradation of myofibrillar proteins in muscle, including troponin I [29, 30], myosin heavy chain [29, 31], myosin‐binding protein C, myosin light chains 1 and 2 [29], and in cultured myotubes also actin [32, 33], whereas atrogin1/MAFbx catalyzes the loss of proteins that promote protein synthesis such as the eukaryotic translation initiation factor 3 subunit f (eIF3‐f) [34]. Another important ubiquitin ligase, Trim32, is expressed across various tissues, and in atrophying muscles it catalyzes the loss of the desmin cytoskeleton and the associated myofibrillar thin filaments (actin, tropomyosin, and troponins), as well as the Z‐line marker α‐actinin [35, 36, 37]. Trim32 also impedes muscle growth by inhibiting the major growth pathway in muscle, insulin‐PI3K‐AKT signaling [38, 39], and can activate autophagy via K63‐linked ubiquitination of the autophagy‐related serine/threonine protein kinase uncoordinated‐51‐like kinase 1 (ULK1) [40, 41]. Additionally, the ubiquitin ligase Tumor necrosis factor receptor‐associated factor 6 (TRAF6) promotes the induction of atrogenes during starvation, glucocorticoid treatment, aging, or denervation [42, 43, 44]; its function is partially mediated by JNK1/2, p38 MAPK, AMP‐activated protein kinase, and NF‐κB [45]. The cellular inhibitor of apoptosis 1 (cIAP1) is another ubiquitin ligase that promotes denervation‐induced atrophy, and its overexpression in myotubes is sufficient to cause wasting [46]. Additional ubiquitin ligases, such as MUSA1 [47], SMART, and Fbxo31 [48], also play prominent roles in atrophy. These ubiquitin ligases clearly cooperate with E2 conjugating enzymes in the ubiquitination and subsequent degradation of muscle proteins during atrophy, and the roles of E2 enzymes in this process have recently become clearer [33, 49, 50, 51, 52].

The excessive breakdown of myofibrillar proteins is preceded and facilitated by the loss of stabilizing systems, such as structural regulatory proteins (e.g., myosin‐binding protein C) [29], the desmin cytoskeleton [35, 53], and structural membrane‐associated complexes [54, 55]. This loss of desmin is primarily mediated by calpain‐1 [35, 56], which belongs to a family of calcium‐dependent cysteine proteases that also include calpain‐2 and the muscle‐specific calpain‐3/p94. These enzymes are activated by elevated intracellular calcium levels, which often result from disrupted calcium homeostasis in states of atrophy such as disuse, denervation, fasting, and cancer [35, 57, 58, 59], due to a decline in SERCA levels [60]. Upon activation, calpain‐1 cleaves the phosphorylated and ubiquitinated desmin filaments, and together with the AAA‐ATPase ATAD1 causes their disassembly and solubilization [36]. As a result, cytoskeletal components, such as desmin filaments and the contractile myofibrils, become more accessible and susceptible to degradation by ubiquitin ligases and the proteasome [61].

## Mechanisms driving fiber‐type transitions during atrophy

Muscle atrophy often triggers shifts in fiber‐type composition, either from fast‐ to slow‐twitch fibers or vice versa, as adaptive responses to various catabolic conditions. Catabolic signals tend to have a greater impact on fast‐twitch glycolytic fibers than on slow‐twitch oxidative fibers. Disuse atrophy, caused by reduced mechanical loading due to immobilization, bed rest, or microgravity, usually leads to a shift from slow‐oxidative (type I) to fast‐glycolytic (type II) [62, 63]. This transition is driven by the reduced activation of oxidative metabolism pathways, diminished mitochondrial biogenesis, and altered calcium signaling [64, 65]. Conversely, aging‐related sarcopenia typically causes a shift from fast‐ to slow‐twitch fibers. Given the greater susceptibility of fast‐twitch fibers to atrophy, methods to activate the transition from fast‐ (type II) to slow‐ (type I) twitch fibers could be of value to combat muscle wasting.

### Increased PGC‐1α activity promotes switching to slow fibers

A promising target to mitigate atrophy is the exercise‐induced transcription coactivator peroxisome proliferator‐activated receptor‐γ coactivator‐1α (PGC‐1α). During exercise, PGC‐1α promotes maintenance of muscle mass and functional capacity by activating AMP‐activated protein kinase (AMPK), sirtuin 1 (SIRT1), calcium/calmodulin‐dependent protein kinase (CaMK), and p38 mitogen‐activated protein kinase (p38 MAPK) [66, 67]. As it is downregulated in various forms of atrophy, maintaining PGC‐1α levels high can counteract various forms of muscle wasting, for example, by inhibiting FOXO and NF‐κB signaling [68, 69, 70]. Furthermore, PGC‐1α reduces proteolysis and oxidative stress in muscles atrophying due to glucocorticoid treatment [71], sepsis [72], chronic kidney disease [73], diabetes [67], and obesity [74]. It was initially identified in brown adipose tissue as a transcriptional coactivator of the peroxisome proliferator‐activated receptor (PPAR)‐γ [75], where it activates the expression of genes that maintain energy homeostasis and mitochondrial biogenesis in response to thermogenesis [76]. In Pompe disease, PGC‐1α expression facilitates a transition from fast‐ to slow‐twitch fibers, which are less prone to atrophy and fatigue [77]. This process involves mitochondrial biogenesis [78], partially by interacting with NRF‐1 [79], activation of oxidative metabolism [80], and increased expression of myofibrillar gene isoforms of oxidative muscle fibers, through myocyte enhancer factor 2 members MEF2C and MEF2D [81]. Moreover, PGC‐1α accumulation may be beneficial for treating atrophy in diabetes by improving insulin sensitivity in insulin‐resistant skeletal muscles [67] and enhancing glucose homeostasis and insulin secretion from pancreatic B cells [82]. Ectopic expression of PGC‐1α in muscle has been shown to improve functional capacity and ameliorate myopathy in *mdx* mouse model for DMD by inducing a fast‐to‐slow fiber‐type shift [83]. Conversely, skeletal muscle‐specific knockout of PGC‐1α caused a transition from slow‐oxidative type I and IIA muscle fibers toward faster type IIX and IIB fibers, reduced endurance capacity, and exacerbated fiber damage and inflammatory markers after treadmill running [84].

One of the various key upstream signals that activate PGC‐1α in muscle during exercise is AMPK, a central regulator of cellular energy homeostasis. When muscle cellular energy charge is low and AMP levels rise (e.g., during fasting), AMPK is activated to restore energy balance by inhibiting anabolic processes like protein synthesis and muscle growth, via suppression of mTORC1 activity [85], either through direct phosphorylation or via the activation of tuberous sclerosis complex 2 (TSC2) [86, 87]. AMPK also activates the eukaryotic elongation factor 2 kinase (eEF2K), which in turn inhibits the elongation factor eEF2 and overall protein synthesis [88]. At the same time, AMPK stimulates proteolysis via both the ubiquitin‐proteasome system (UPS) and autophagy [86, 88, 89], partly by activating ULK1 [89] and by inducing FOXO3‐mediated atrogenes expression [90, 91]. Accordingly, transgenic expression of a dominant‐negative inhibitor of AMPK in muscle was sufficient to reduce MuRF1 expression, ubiquitin conjugate levels, and muscle weight loss in slow‐twitch soleus muscle during disuse atrophy caused by unloading [92].

Prolonged treatment with the AMPK analog, 5‐aminoimidazole‐4‐carboxamide riboside (AICAR), increased IGF‐1 and PGC‐1a levels in mouse muscle, improving aerobic metabolism and the resistance to fatigue upon running [93] while attenuating atrophy (reduced atrogene expression) during aging [94] or inflammation [95]. However, no beneficial effects of this drug were observed on muscle wasting following denervation [68]. Activation of AMPK signaling by L‐leucine administration increased slow‐fiber‐related genes in rat skeletal muscles [96], and in horses, induced slow‐MYH expression, enhanced mitochondrial biogenesis, and stimulated the proliferation of satellite cells [97, 98]. These findings about satellite cell proliferation suggest that leucine may be useful for treating muscle wasting associated with impaired muscle regeneration, such as in muscular dystrophies, sarcopenia, and injury‐related muscle degeneration. Furthermore, the SIRT1 activator, resveratrol, has been shown to induce fast‐to‐slow fiber‐type transition by activating AMPK/PGC‐1α signaling and mitochondrial biogenesis in bovine myotubes [99]. Nutrients like arginine can also facilitate the transition from fast‐to‐slow fibers via the SIRT1/AMPK pathway [100].

### Activation of calcium signaling promotes transition to slow‐twitch fibers

The calcium‐dependent serine/threonine protein phosphatase, calcineurin, which has been implicated in the maintenance of skeletal muscle mass, plays a critical role in fast‐to‐slow muscle fiber‐type switching [101, 102, 103]. Calcineurin is a heterodimer complex consisting of a catalytic A subunit, which binds the calcium‐binding protein calmodulin, and a smaller calcium‐binding regulatory B subunit [104]. Upon activation, calcineurin interacts with and dephosphorylates the N‐terminal conserved regulatory domain of the transcription factor NFAT, which subsequently translocates into the nucleus and associates with other transcription factors, including MEF2 [105, 106], to activate the expression of slow‐twitch fiber genes, including PGC‐1α [107]. Transgenic expression of calcineurin in mouse skeletal muscles mimics the effects of PGC‐1α overexpression by promoting fast‐to‐slow fiber‐type transition [81, 108, 109]. Furthermore, in rats, activation of calcineurin/NFAT signaling by the anabolic steroid nandrolone protects from atrophy on denervation [110]. Conversely, reduced calcineurin expression or activity correlates with decreased PGC‐1α expression and atrophy (e.g., in diabetes) [111].

### FOXO1 promotes transition to fast‐twitch fibers, which are susceptible to atrophy

The balance between muscle growth and atrophy depends primarily on the activity of PI3K‐AKT–mTOR signaling [112]. In muscle, this pathway activates overall protein synthesis and reduces protein degradation by inhibiting FOXO transcription factors [113]. In various forms of muscle atrophy (e.g., fasting, untreated diabetes, obesity, and immobilization), reduced PI3K–AKT–mTOR activity leads to dephosphorylation of FOXO3, which in turn translocates into the nucleus and activates the expression of genes that promote proteolysis via the UPS and autophagy [114, 115]. In slow‐twitch fibers, PGC‐1α prevents atrophy in part by inhibiting the association of FOXO3 with the promoters of its target genes [70]. However, fast‐twitch fibers have lower levels of PGC‐1α, rendering them more susceptible to FOXO3‐mediated atrophy [116]. Interestingly, FOXO1, another member of the FOXO family, promotes a shift toward fast‐twitch fibers by suppressing muscle oxidative metabolism, partly through inhibition of calcineurin signaling [117]. Overexpression of FOXO1 in C2C12 myotubes reduces the levels of myogenin transcription factors, which are primarily expressed in slow‐oxidative fibers [117], calcineurin phosphatase activity, and the expression of modulatory calcineurin interacting protein, exon 4 isoform MCIP1.4 in neonatal cardiomyocytes [118]. Consequently, methods to reduce FOXO1 activity may help increase the proportion of slow‐twitch muscle fibers, which are more resistant to atrophy.

### Reduced NF‐κB signaling protects fast‐twitch fibers from atrophy

NF‐κB is another transcription factor associated with muscle atrophy, and its inhibition has been shown to reduce wasting, particularly in fast‐twitch muscle fibers [119]. In NF‐kB null mice, atrophy upon hindlimb unloading is less pronounced in fast‐twitch fibers compared with slow‐twitch fibers [120]. In addition, inhibiting NF‐κB activity in mouse muscles through the expression of IκB significantly reduced muscle atrophy following denervation in tumor‐bearing mice [121] or fasting [122]. Similarly, mice deficient in IκB kinase‐β (IKK2) exhibited reduced atrophy after denervation [119]. The precise mechanisms by which NF‐κB contributes to muscle atrophy remain largely unclear, although TRAF6 [43] and tumor necrosis factor‐like weak inducer of apoptosis (TWEAK) are likely to be important [123]. Increased calcium influx into muscle, in part due to elevated membrane content of hemichannels (formed by connexins) and increased sarcolemma permeability, has been shown to activate NF‐κB and the expression of proinflammatory genes [124, 125]. This mechanism contributes to muscle atrophy in DMD [124], and in denervated fast‐twitch muscles [126]. Thus, inhibiting NF‐κB or reducing hemichannels activity could be valuable strategies to combat muscle wasting, especially in conditions like DMD and denervation. One potential approach is the activation of PGC1‐α, which suppresses FOXO‐ and NF‐κB‐dependent transcription during fasting‐ or denervation‐induced atrophy [68, 69]. This strategy may be particularly effective in preventing wasting of fast‐twitch fibers, where PGC1‐α levels are normally low. In contrast, slow‐twitch fibers likely maintain lower NF‐κB activity due to inherently higher PGC1‐α expression.

## Metabolic reprogramming as a strategy to prevent atrophy

Metabolic reprogramming contributes to muscle atrophy by facilitating the degradation of myofibrillar and soluble proteins. Upon unloading or disuse (e.g., immobilization, bedridden patients, microgravity) of skeletal muscle, the reduced energy utilization is accompanied by the accumulation of calcium and ROS, and a decrease in nitric oxide (NO) content [127, 128, 129]. These changes lead to atrophy of especially slow‐twitch fibers [127, 129, 130]. Histologically, unloading due to prolonged bed rest or spaceflight induces a transition from slow to fast fibers [131, 132], which are more prone to atrophy, ultimately leading to frailty and reduced strength [133, 134]. Under these conditions, mTOR signaling decreases, and consequently, protein synthesis falls; simultaneously, protein degradation increases largely via FOXO‐mediated expression of the atrogene program. The induction of the major atrogenes, UPS components, and the ubiquitin ligases MuRF1 and atrogin1/MAFbx represents an early response to unloading and other types of atrophy.

Cellular calcium homeostasis is also disrupted during disuse and aging, due to reduced SERCA activity, increased connexin hemichannels, and impaired calcium pumping out of the cell [126, 135, 136]. The resulting elevated cellular calcium levels activate calcium‐specific proteases such as calpain‐1, which contribute to atrophy partly by promoting the disassembly of the desmin cytoskeleton [35, 36, 137]. Additionally, hindlimb suspension in rats decreases neuronal nitric oxide synthase (nNOS) activity and alters its cellular distribution, leading to reduced NO levels in soleus muscle [127]. Consequently, proteolysis via GSK‐3β is accelerated, and the expression of slow‐MYH isoform falls [138].

Interestingly, interventions that restore NO levels help preserve slow‐twitch fibers. L‐arginine administration has been shown to maintain NO production, restore slow‐MYH mRNA expression, and enhance PGC‐1α and calcineurin/NFATc1 activity, consequently preserving the slow‐twitch fiber phenotype [139]. Similar findings have been reported in humans following prolonged bed rest or space missions, where the expression of slow‐MYH‐I isoform declines while fast MYH‐IIA and MYH‐IIX isoforms increase, suggesting a shift from oxidative to glycolytic metabolism [140, 141, 142, 143]. This decrease in muscle oxidative capacity is accompanied by reduced mitochondrial density and a decline in metabolic enzymes of the tricarboxylic acid cycle (TCA) and β‐oxidation pathways [64]. Conversely, in conditions of negative energy balance (e.g., caloric restriction or fasting) there is a preferential shift from fast‐ to slow‐twitch fibers [144, 145]. This metabolic adaptation suggests that targeted interventions that maintain slow‐twitch fiber content, enhance mitochondrial function, and preserve NO signaling could be effective in combating disuse atrophy.

## Pathways promoting transition to oxidative fibers

Muscle atrophy remains a major clinical problem, and currently there are no effective FDA‐approved therapies. While substantial progress has been made in our understanding of the molecular mechanisms driving atrophy, effective treatments must not only increase muscle mass but also preserve fiber strength, composition, and metabolic function. Several promising drug targets have been identified, and new therapies for muscle wasting are entering clinical trials; though long‐term efficacy and safety remain key considerations. Currently, the only validated treatment is exercise, which effectively counteracts skeletal muscle wasting by promoting oxidative metabolism and fiber‐type transitions [146]. It is known to induce PGC‐1α expression [67, 147], which protects against various forms of atrophy [68, 69]. However, for patients who are bedridden, frail, immobilized, or ventilated, exercise is impractical, and alternative strategies are necessary to mimic exercise‐induced metabolic benefits.

### PGD2 signaling and fiber‐type regulation

The lipid PGD2 (Prostaglandin D) and its receptor DP2 have emerged as key regulators of fiber‐type composition in muscle wasting, particularly in conditions like obesity and DMD [148, 149]. Elevated PGD2 levels in the muscles of mice fed a high‐fat diet (HFD) suppress the transition from glycolytic to oxidative muscle fibers. Depletion of DP2 in mice, either through skeletal muscle‐specific gene deletion or by the injection of the selective inhibitor CAY10471, was sufficient to cause a shift toward oxidative fibers, improve muscle endurance, reduce adiposity, mitigate hepatic lipid accumulation, and improve insulin sensitivity. These beneficial effects seemed to require the nuclear translocation of NFATc1 because DP2 activation, as occurs in muscles from obese or *mdx* mice, prevents NFATc1's nuclear entry via the RhoA/ROCK2 signaling and blocks the transition to slow‐twitch fibers [149]. Given that NFATc1, which is also activated by muscle contraction, is pivotal for the transition from fast‐ to slow‐twitch muscle fibers, its activation may provide a therapeutic target for preserving oxidative muscle capacity in atrophy [150]. Additionally, endurance training promotes retention of slow‐twitch fibers by reducing FoxO1‐dependent transcription [117, 151].

### Dietary interventions to maintain oxidative fiber composition

Dietary compounds have been shown to influence muscle fiber‐type composition, favoring a shift toward oxidative metabolism and maintenance of slow‐twitch fibers. Arginine treatment induces slow MYH, increases SIRT1 and PGC‐1α levels, while concomitantly reducing fast MYH expression [100]. This shift is accompanied by increased activity of several oxidative enzymes, including SDH and MDH, and decreased activity of the glycolytic enzyme LDH, both in mice and cultured C2C12 myotubes [100]. Similarly, ferulic acid (FA) improves skeletal muscle endurance capacity by driving transformation to oxidative fibers [152] via AMPKα2 and PGC‐1α activation [152]. Additional compounds with similar effects include Oleanolic acid, which acts through TGR5‐mediated CaN/NFATc1 signaling [153], and apple polyphenols, which induce slow MYH in rat hindlimb muscles [154]. Dietary butyrate, a short‐chain fatty acid, stimulates mitochondrial biogenesis and PGC‐1α expression, leading to increased content of slow‐twitch fibers in porcine skeletal muscles [155]. Another compound is garcinol, extracted from *Garcinia indica* fruit peel, which enhances transformation to oxidative fibers through PGC‐1α‐dependent mechanism [156]. These findings suggest that targeted nutritional strategies may potentially be useful for modulating muscle fiber composition to enhance oxidative capacity.

### Pharmacological strategies for metabolic reprogramming

Several pharmacological agents have demonstrated potential in promoting oxidative muscle metabolism and preserving slow‐twitch fibers. The exercise mimetic, Eugenol, enhances endurance capacity, induces a muscle fiber shift from fast to slow, and promotes white fat browning and lipolysis via TRPV1‐mediated CaN signaling [157]. Similarly, AMPK activators, such as AICAR, induce muscle fiber switching toward the oxidative phenotype in rat skeletal muscle [158]. Metformin, another AMPK activator, mitigates disuse atrophy in the soleus muscle by preventing the transition from slow to fast‐twitch fibers during hindlimb suspension [159]. Additionally, PPARα agonists like fenofibrate enhance the content of type I and IIA fibers, promoting a slow, more oxidative phenotype in *mdx* mice [160]. Chronic administration of resveratrol further promotes a slow, oxidative fiber profile in *mdx* mice, likely through PGC‐1α activation [161].

## Conclusions and future directions

Promoting a shift toward oxidative, slow‐twitch muscle fibers may present a promising strategy for improving metabolic health and attenuating muscle wasting. The high oxidative capacity of slow‐twitch fibers plays a vital role in improving endurance, insulin sensitivity, and overall energy metabolism. Because exercise remains the most effective approach for maintaining muscle mass and oxidative capacity, alternative strategies that mimic its benefits are essential for treating patients who are unable to exercise, including bedridden, immobilized, sedated, or ventilated individuals. However, when designing therapies aimed at inducing fiber‐type transitions, it is important to consider the inherent differences in fiber size between species. In humans, where the different fiber types have similar cross‐sectional areas, shifting toward slow‐twitch fibers may primarily enhance endurance and metabolic health without markedly reducing muscle mass [1, 5]. In contrast, in rodents, where fast‐twitch fibers are much larger, such a shift could result in a noticeable reduction in muscle size and a consequent loss of strength [5]. These differences emphasize the need for careful evaluation of muscle function, not just fiber composition, when translating preclinical findings into therapeutic applications.

Substantial progress has been made in our understanding of the structural, metabolic, and biochemical characteristics of muscle fiber types; yet, the molecular mechanisms governing fiber‐type transitions and their impact on atrophy are only beginning to be understood. Potential approaches to induce a metabolic shift toward oxidative fibers include enhancing PGC1‐a, AMPK (e.g., by AICAR and metformin), calcineurin/NFAT pathway, SIRT1 (e.g., using resveratrol), PPAR‐a (e.g., using its agonists) and NO signaling, and/or inhibiting FOXO1 and PGD2 signaling (Table 3). An additional compelling target is HDAC4 signaling, as its inhibition in skeletal muscle using shRNA preserves slow‐twitch fiber composition, reduces atrophy‐related gene expression, and enhances PGC‐1α and SIRT1 levels, key regulators of mitochondrial biogenesis and oxidative capacity [162]. In parallel, protecting type II fibers from atrophy is critical for muscle strength (e.g., by the inhibition of NF‐kB).

**Table 3:** Pharmacological targets to treat muscle fiber atrophy.

Catabolic state	Strategy	Expected effect	Ref.
Pompe disease	↑PGC‐1α	↑Type I fibers	[77]
DMD	↑ PGC‐1α ↑ SIRT1 ↑ PPAR‐α ↓ NF‐κB	↑ Type I fibers ↓ Myopathy	[83] [124]
Diabetes	↑ AMPK ↑ PGC‐1α ↑ Calcineurin	↑Type I fibers ↑Insulin sensitivity	[67] [111]
Denervation	↑Calcineurin/NFAT ↓ NF‐κB	↓Fiber atrophy	[110] [121]
Fating/malnutrition	↓ NF‐κB Feeding	↓Fiber atrophy	[122]
Obesity	↓ PGD2	↑Insulin sensitivity ↑Type I fibers	[149]
Sarcopenia	↑ AMPK ↑ PGC‐1α	Preserve type II fibers	[94]
Disuse/immobilization/bed rest	↑ AMPK ↓ NF‐κB ↑ mTOR activity ↑ NO	Preserve type I fibers ↑Type I fibers	[138, 159]

A major challenge in the field of muscle atrophy and disease is the delivery of therapeutic agents directly into muscle tissue. Nutritional interventions that can protect from atrophy and/or promote transition toward slow‐twitch fibers, such as specific dietary supplements, offer an advantage in bypassing this hurdle, provided they naturally enter circulation and are readily consumed by muscle under normal physiological conditions. For other pharmacological agents, such as AMPK activators, emerging innovative approaches such as skeletal muscle‐derived exosomes hold promise, although their use as therapeutic vehicles requires further investigation.

Advancing our understanding of fiber‐type‐specific mechanisms will facilitate the development of new treatment strategies, such as nutritional interventions, pharmaceutical agents, combined with specific exercise regimens, to preserve muscle mass and function in various clinical conditions. However, a balanced approach is crucial, as fast‐twitch fibers are indispensable for strength, power, and mobility, especially in aging populations where maintaining these functions is vital for overall health and independence. A future approach that optimally supports both fiber types may be the most effective strategy for muscle health management across various conditions, from aging to disease.

## Conflict of interest

The authors declare no conflict of interest.

## Author contributions

S.C., R.B.‐D., and S.S. wrote the paper.
